# The polymorphism rs35767 at *IGF1* locus is associated with serum urate levels

**DOI:** 10.1038/s41598-018-29665-3

**Published:** 2018-08-16

**Authors:** Gaia C. Mannino, Anastasia Fuoco, Maria A. Marini, Rosangela Spiga, Concetta Di Fatta, Elettra Mancuso, Francesco Perticone, Francesco Andreozzi, Giorgio Sesti

**Affiliations:** 1 Department of Medical and Surgical Sciences, University “Magna Graecia” of Catanzaro, Catanzaro, Italy; 2 Department of Systems Medicine, University of Rome-Tor Vergata, Rome, Italy

## Abstract

Previous studies suggested that the IGF-1/IGF-1 receptor signaling pathway may contribute to regulate uric acid levels. To confirm this hypothesis, we assessed the effects of the IGF-1-raising genetic variant rs35767 on urate levels in serum and urine, and we investigated IGF-1 ability to modulate the expression of transporters involved in reabsorption and secretion of uric acid in the kidney. The study population included 2794 adult Whites. 24-hour urinary uric acid concentration was available for 229 subjects. rs35767 polymorphism was screened using TaqMan genotyping assays. HEK293 (human embryonic kidney-293) cell line was treated with IGF-1 (1, 5, 10, 50 nM) for 24-hours, and differences in the expression of urate transporters were evaluated via Western Blot and real time rtPCR. Individuals carrying the IGF-1-raising allele (rs35767 T) exhibited significantly lower levels of serum urate according to both additive and recessive models, after correction for gender, age, BMI, glucose tolerance, glomerular filtration rate, and anti-hypertensive treatment. TT genotype carriers displayed higher uricosuria than C allele carriers did, after adjusting for confounders. Exposure of HEK293 cells to IGF-1 resulted in a dose-dependent increase of uric acid transporters deputed to uric acid excretion (MRP4, NPT1 and BCRP), and reduction of GLUT9 expression, the major mediator of uric acid reabsorption, both at mRNA and protein level. We observed a significant association between the functional polymorphism rs35767 near *IGF1* with serum urate concentrations and we provide a mechanistic explanation supporting a causal role for IGF-1 in the regulation of uric acid homeostasis.

## Introduction

Uric acid is a final breakdown product of purine degradation and circulates in the blood as urate. Concentrations of serum urate are increasing worldwide, owing in part to dietary and lifestyle factors, and rising prevalence of obesity, metabolic syndrome, impaired glucose tolerance/type 2 diabetes, insulin resistance, chronic kidney disease, and cardiovascular diseases, all of which are associated with hyperuricemia^1–11^. Serum urate levels depend on the homeostasis between uric acid production, mainly from purine degradation in the liver, and its removal via the kidney and gut. Elevated serum urate levels are attributable to the net reabsorption of filtered uric acid in the proximal tubules^12^. In addition to the environmental factors, it has been estimated that heritability accounts for 40−70% of serum urate concentration variability^13,14^. A number of genome-wide association studies (GWAS) have so far identified 28 genomic loci associated with urate concentrations at genome-wide level of significance, and gout^15–23^. Notably, one of these loci associated with uric acid concentrations and gout was the intronic rs6598541 SNP located within *IGF1R* encoding for the receptor of insulin-like growth factor 1 (IGF-1)^22,23^, and the allele of the rs6598541 SNP associated with higher serum urate concentrations (0.043 mg/dl increase [CI 0.031–0.055], *P* = 5 × 10^−15^) was also found to be associated with lower fractional excretion of uric acid (*β* = −0.059% increase [s.e. = 0.019], *P* = 1.5 × 10^–3^)^22^. Interestingly, we have shown an inverse relationship between circulating IGF-1 and urate levels in nondiabetic adult subjects^24^ consistent with a prior pilot experimental study showing that IGF-1 acutely decreases serum urate concentration^25^, thus suggesting that the IGF-1/IGF-1 receptor signaling pathway plays a role in regulating serum urate levels.

In order to verify this hypothesis, we decided to assess the association of a functional polymorphism (rs35767) located within the promoter of the *IGF1* gene with urate levels in adult subjects of European ancestry. Prior studies have demonstrated that individuals carrying the T allele at rs35767 exhibit increased levels of circulating IGF-1 as compared with CC carriers^26–31^. Therefore, we interrogated the NHGRI-EBI GWAS Catalog database^32^ and we consulted the publicly accessible collection of complete results from the network analysis accomplished by Köttgen A. *et al*. in December 2012^22^ to investigate if this polymorphisms affecting *IGF-1* gene expression was associated with serum urate levels. Two polymorphisms in close proximity with rs35767, rs35765 and rs855214 (each scoring r^2^ = 0.74 in the CEU population of the HapMap project), were nominally associated with serum urate levels (*P* = 0.013 and *P* = 0.026, respectively), although this association did not reach genome-wide significance (*P* < 5 × 10^−8^).

To further clarify the impact of rs35767 on serum urate levels, we addressed its effects on circulating urate concentrations by screening a large cohort of well-characterized adult White Europeans. One potential explanation for how IGF-1/IGF-1 receptor signaling pathway might contribute to regulate serum urate levels might be its ability to modulate expression of transporters involved in reabsorption and secretion of uric acid in the kidney. Therefore, we examined the *in vitro* effect of IGF-1 on renal expression of uric acid transporters in Human Embryonic Kidney (HEK293) cells.

## Materials and Methods

### Study subjects

We analyzed 2794 unrelated individuals consecutively recruited at the Department of Medical and Surgical Sciences of the University “Magna Graecia” of Catanzaro and at the Department of Systems Medicine of the University of Rome-Tor Vergata as previously described^33,34^. The criteria for recruitment were: presence of at least one cardio-metabolic risk factor including but not limited to family history of diabetes, dysglycemia/type 2 diabetes, hypertension, dyslipidemia, and overweight/obesity. Patients were excluded from the study if they had: gout, history of malignant diseases, end stage renal disease, heart failure, gastrointestinal affections with bleeding or malabsorption, anemia, hemoglobinopathies, hemolytic disease, autoimmune disorderss, acute or chronic infections, pancreatitis, accumulation diseases such as amyloidosis and hemochromatosis, history of drug abuse, self-reporting alcohol consumption of >20 g⁄day, positivity for antibodies to hepatitis C virus (HCV) or hepatitis B surface antigen (HBsAg), and corticosteroids use.

All patients were seen in the morning, after an overnight fast. In these circumstances information concerning medical history, drug use, and alcohol consumption were collected, anthropometrical parameters were recorded (i.e. body mass index (BMI), waist circumference, clinic blood pressure, etc.) and a sample of venous blood was drawn for laboratory determinations. A 75 g OGTT was carried out in subjects with no history of type 2 diabetes with sampling for plasma glucose determination. Twenty-four-hour urinary uric acid concentrations were measured concurrently on the day of blood sampling in 229 individuals.

The study was approved by the Institutional Ethics Committees of the University Magna Graecia of Catanzaro and the University of Rome Tor Vergata. All patients signed a written informed consent prior entering the study, in accordance with the principles of the Declaration of Helsinki.

### Biochemical determinations

Serum urate levels were measured by the URICASE/POD method implemented in an autoanalyzer (Boehringer Mannheim, Mannheim, Germany). Glucose concentrations were measured by enzymatic methods (Hoffman-La Roche, Basel, Switzerland). Glycated hemoglobin (HbA1c) was assessed by high performance liquid chromatography employing a National Glycohemoglobin Standardization Program (NGSP) certified automated analyzer (Adams HA-8160 HbA1C analyzer, Menarini, Italy). Serum creatinine concentrations were assessed by an automated technique based on a Creatinine Jaffè compensated method for serum (Roche Diagnostics) implemented in an auto-analyzer.

### rs35767 genotyping

Genomic DNA was extracted from whole peripheral blood using a commercial DNA purification kit (Promega, Madison, WI). rs35767 genotype calls were determined with TaqMan allelic discrimination assay (Assay ID# C__799146_10; Applied Biosystems, Foster City, CA). Template DNA was amplified and fluorescence was detected on an iCycler Thermal Cycler with iQ5 Multicolor Real-Time PCR Detection System (Bio-Rad Laboratories, Inc., Hercules, CA). Good genotyping quality was ensured by including 3 HapMap samples in each 96- well plate. Genotyping concordance of HapMap controls was >99%.

### Cell culture and treatment

HEK293 (human embryonic kidney cell line) were grown in complete high glucose Dulbecco’s Modified Eagles’s Medium (DMEM, Sigma-Aldrich, Milan, Italy) supplemented with 10% (vol/vol) fetal bovine serum and 1% (vol/vol) penicillin/streptomycin, and maintained in a humidified atmosphere of 5% CO_2_ in air at 37 °C. For experiments cells were treated with IGF-1 (Sigma-Aldrich, Milan, Italy) at different concentration (1, 5, 10, 50 nM) for 24 h.

### RNA extraction and real time PCR

After IGF-1 treatment, total RNA was extracted from HEK293 by Trizol reagent (Life Technologies, Gaithersburg, MD, USA) and reverse transcribed using the High Capacity cDNA Reverse Transcription Kit (Applied Biosystems, Foster City, CA). mRNA expression of *ABCG2* (BCRP, Hs01053790_m1), *ABCC4* (MRP4, Hs00988717_m1), *SLC17A1* (NPT1, Hs00192656_m1), *SLC2A9* (GLUT9, Hs00417125_m1), *SLC22A11* (OAT4, Hs00945829_m1) and *SLC22A12* (URAT1, Hs00375985_m1) was analyzed by quantitative Real-Time PCR technique using TaqMan pre-designed gene-expression assays (Thermo Fisher Scientific, Waltham, MA, USA) on iQ5 real-time thermocycler (Bio-Rad, Hercules, CA, USA). Results were normalized to the housekeeping gene *18S* (Hs03928989_g1). All reactions were performed in triplicate. Relative gene expression was calculated by using the comparative C_T_ (Livak) method. All reagents were obtained from Life Technologies Italia (Monza, Italy).

### Western blot analysis

HEK293 proteins were extracted by lysing cells in buffer containing 50 mmol/L HEPES (pH 7.5), 150 mmol/L NaCl, 10 mmol/L EDTA, 1% Triton X-100, 10 mmol/L Na4P2O7, 100 mmol/L NaF, and 2 mmol/L sodium orthovanadate supplemented with protease inhibitors cocktail. Protein concentration was determined with the Bradford assay (DC Protein Assay; Bio-Rad, Hercules, CA) according to the manufacturer’s instructions.

Equal amounts of proteins were resolved by SDS-PAGE and electrotransferred to nitrocellulose membrane (Amersham Biosciences, Piscataway, NJ). Membranes were blocked with TBS with 5% milk according to antibodies’ producers instructions, then probed with primary antibodies followed by incubation with peroxidase-conjugated secondary antibodies. Proteins were detected by using enhanced chemiluminescence (Amersham Biosciences, Piscataway, NJ), and band densities were quantified by densitometry using ImageJ software. To normalize the blots for protein levels, after being immunoblotted with specific antibodies, the membranes were probed with beta-actin antibody.

### Reagents

HEK293 were purchased from ATCC (American Type Culture Collection, Manassas, USA). Media, sera and antibiotics for cell culture were from Sigma (Walkersville, MD, USA). Protein electrophoresis and western blot reagents were from Bio-Rad (Richmond, VA, USA) and electrochemiluminescence reagents from Pierce (Rockford, IL, USA). IGF-1 was purchased from Thermo Fisher Scientific. The antibodies used were: anti-BCRP (*ABCG2-ATP binding cassette subfamily G member 2*) (Abbiotec, LCC, Dunbrook Rd, Ste A, San Diego, USA), anti-NPT1 (*SLC17A1-solute carrier family 17 member 1*) (Millipore, Burlington, Massachusetts, Stati Uniti), anti-MRP4 (*ABCC4-ATP binding cassette subfamily C member 4*) (Abcam, Cambridge, UK), anti-GLUT9a (*SLC2A9-solute carrier family 2 member 9*, *protein isoform 1*) (Novus Biologicals, Littleton, Colorado, USA), anti-OAT4 (*SLC22A11-solute carrier family 22 member 11*) (Abcam, Cambridge, UK), anti-URAT1 (*SLC22A12-solute carrier family 22 member 12*) (Abnova, Taiwan) and β-actin (R&D Systems, Minneapolis, Minnesota, USA).

### Calculations

Study participants were categorized in accordance with their glucose tolerance status as NGT (normal glucose tolerance) when their fasting plasma glucose and 2-h post-challenge levels were <126 mg/dl and <140 mg/dl, respectively; IGT (impaired glucose tolerance) when fasting plasma glucose was <126 mg/dl and 2 h post-load was 140–199 mg/dl; type 2 diabetes when fasting plasma glucose was ≥126 mg/dl and/or 2 h post-load was >200 mg/dL. BMI was calculated as the ratio of weight in kg to the square of height in m. The MDRD equation [eGFR = 175 × (serum creatinine)^−1.154^ × (Age)^−0.203^ × (0.742 if female)] was employed to estimate glomerular filtration rate (eGFR)^35^.

### Statistical analysis

Continuous variables are summarized as means ± SD. Comparisons of continuous variables within genotype groups were adjusted for confounding factors such as age, gender and BMI by ANCOVA (general linear model). Categorical variables were compared by χ^2^-test. In order to estimate the independent contribution of the rs35767 polymorphism to serum urate levels, we performed a linear regression analysis in a model which also included gender, age, and BMI. Genotype distributions were in Hardy Weinberg equilibrium (P > 0.05). Power calculations were performed with Quanto version 1.2.4 (http://hydra.usc.edu/gxe; accessed 25 July 2011). The study had 98% power (for α = 0.05) to detect a 0.2 mg/dL change in serum urate levels per allele T. All tests were two-sided, and a P value < 0.05 was considered statistically significant. All analyses were performed using SPSS 22.0 (Chicago, IL, USA) software for Windows.

### Data availability statement

All relevant data, materials and information is included in the manuscript.

## Results

Table 1 summarizes anthropometrical and biochemical parameters of our population according to the genotype at rs35767. The association of the variant rs35767 with patients’ clinical features was analyzed according to an additive and a recessive genetic model (Table 1). The distribution of age, gender, BMI, waist circumference, systolic and diastolic blood pressure, fasting and 2 h post-load plasma glucose, HbA1c, glucose tolerance status, and eGFR was not significantly affected by rs35767 polymorphism (Table 1). Conversely, the rs35767 polymorphism exhibited a significant association with serum urate after adjusting for age, gender and BMI in either genetic models (Table 1). Notably, the association between the rs35767 polymorphism and serum urate concentrations remained significant after further adjusting for glucose tolerance status, and eGFR in addition to age, gender and BMI in both an additive (*P* = 0.05) and a recessive (*P* = 0.02) genetic model. We observed no statistical differences amongst genotype groups in the frequency of anti-hypertensive treatment known to affect urate serum concentration, including diuretics, beta-blockers, calcium channel blockers, angiotensin receptor blockers, and ACE inhibitors. Accordingly, the association observed between rs35767 genotype and serum urate concentrations remained significant after adjusting for anti-hypertensive treatments in addition to age, gender and BMI in both an additive (*P* = 0.03) and a recessive (*P* = 0.01) genetic model. To estimate the independent contribution of the rs35767 polymorphism to serum uric acid levels, we performed a linear regression analysis in a model which also included gender, age, and BMI. Comparison of standardized coefficients allowed the determination of the relative strength of each trait’s association with serum uric acid levels (listed from strongest to weakest): male gender (β = 0.397, P < 0.0001), BMI (β = 0.262, P < 0.0001), age (β = 0.132, P < 0.0001), and the rs35767 polymorphism (β = −0.04, P = 0.03).

**Table 1:** Clinical characteristics of 2794 study subjects according to the SNP rs35767 near *IGF1*.

Variables	CC	CT	TT	*P*_additive model_ (C/C vs. C/T vs. T/T)	*P*_*recessive model*_ (C/C + C/T vs. T/T)
Female/Male	914/895	436/423	63/63	0.98	0.99
Age (yr)	49.8 ± 13.8	50.4 ± 14.3	50.6 ± 11.4	0.57^§^	0.63^§^
BMI (kg/m^2^)	30.2 ± 6.4	29.9 ± 6.5	30.0 ± 6.5	0.53*	0.97*
Waist circumference (cm)	100.6 ± 14.8	100.3 ± 15.4	101.1 ± 15.5	0.86*	0.77*
Fasting glucose (mg/dL)	105 ± 35	108 ± 40	104 ± 35	0.06	0.39
2h-plasma glucose (mg/dL)	128 ± 43	127 ± 44	126 ± 47	0.90	0.69
HbA1c (%) [mmol/mol]	5.9 ± 1.2 [41]	6.0 ± 1.5 [42]	6.0 ± 1.3 [41]	0.39	0.87
Systolic blood pressure (mmHg)	130 ± 18	131 ± 18	130 ± 16	0.61	0.32
Diastolic blood pressure (mmHg)	80 ± 11	80 ± 10	80 ± 10	0.74	0.69
Creatinine (mg/dl)	0.81 ± 0.24	0.81 ± 0.24	0.77 ± 0.15	0.13	0.06
eGFR (ml/min/1.73 m^2^)	100 ± 23	99 ± 24	101 ± 19	0.45	0.22
Uric acid (mg/dL)	5.21 ± 1.41	5.16 ± 1.36	4.90 ± 1.37	0.02	0.007
Urinary uric acid (mg/dL) (No. 229 subjects)	37.9 ± 25.8 (No. = 152)	33.6 ± 26.46 (No. = 68)	59.1 ± 39.8 (No. = 9)	0.06	0.03
NGT/IFG/IGT/combo IFG + IGT/T2DM (No.)	901/205/171/131/401	416/89/75/65/214	61/18/10/11/26	0.75	0.73
ACE inhibitor therapy, No.(%)	365 (20.2%)	181 (21.1%)	31 (24.6%)	0.46	0.19
Angiotensin receptor blocker therapy, No.(%)	298 (16.5%)	151 (17.6%)	15 (11.9%)	0.27	0.18
Diuretics, No.(%)	320 (17.7%)	163 (19.0%)	17 (13.5%)	0.30	0.18
Calcium channel blockers, No. (%)	243 (13.4%)	112 (13.0%)	18 (14.3%)	0.91	0.75
Beta blockers, No. (%)	284 (15.7%)	141 (16.4%)	17 (13.5%)	0.68	0.81

Data are as means ± SD. Categorical variables were compared by χ^2^ test. Comparisons between the groups were performed using a general linear model. *P* values refer to results after analyses with adjustment for age, gender, and BMI. ^§^*P* values refer to results after analyses with adjustment for gender. *P values refer to results after analyses with adjustment for gender, and age.

Twenty-four-hour urinary uric acid concentrations were available for 229 individuals (Table 1). According to a recessive genetic model, homozygous carriers of the minor T allele exhibited significantly higher uricosuria in comparison to carriers of the major C allele, after correction for age, gender and BMI (Table 1). This association remained significant (*P* = 0.04) after further adjusting for glucose tolerance status, eGFR, and anti-hypertensive treatments in addition to age, gender and BMI.

Taken together, these data support our hypothesis that the IGF-1-raising rs35767 polymorphism may contribute to the modulation of serum urate levels by enhancing excretion of uric acid. Therefore, we hypothesized that IGF-1 might modulate the expression of uric acid transporters involved in bidirectional renal uric acid handling. To address this issue an *in vitro* model of Human Embryonic Kidney (HEK293) cells, was used for further experiments.

### Effects of IGF-1 on mRNA expression and protein levels of transporters involved in reabsorption and secretion of uric acid in HEK293 Cells

**Figure 1:**
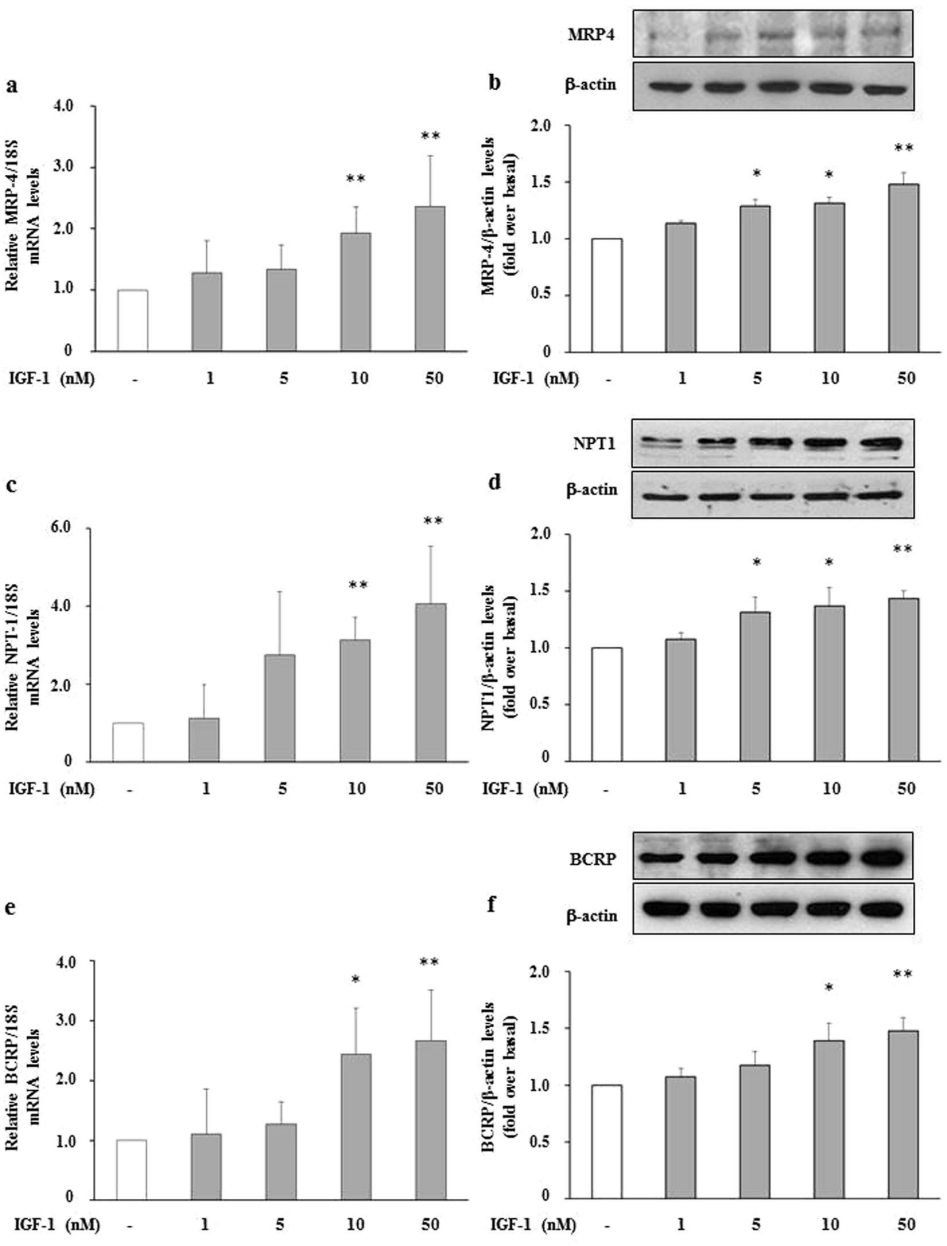
Effect of IGF-1 on urate excretory transporters, mRNA and protein levels. HEK293 cells were incubated in presence of increasing IGF-1 concentrations (5, 10, 50 nM). mRNA levels were measured by Real Time RT-PCR. **(a)** MRP4 mRNA levels; **(b)** MRP4 protein levels; **(c)** NTP1 mRNA levels; **(d)** NPT1 protein levels; **(e)** BCRP mRNA levels; **(f)** BCRP protein levels. Data are means ± SD of three independent experiments, each done in triplicate. *P < 0.02 and **P < 0.01 vs. basal. To see the original blots from which these images were taken, please refer to the Online Supplementary Information (Supplemental Fig. 1).

**Figure 2:**
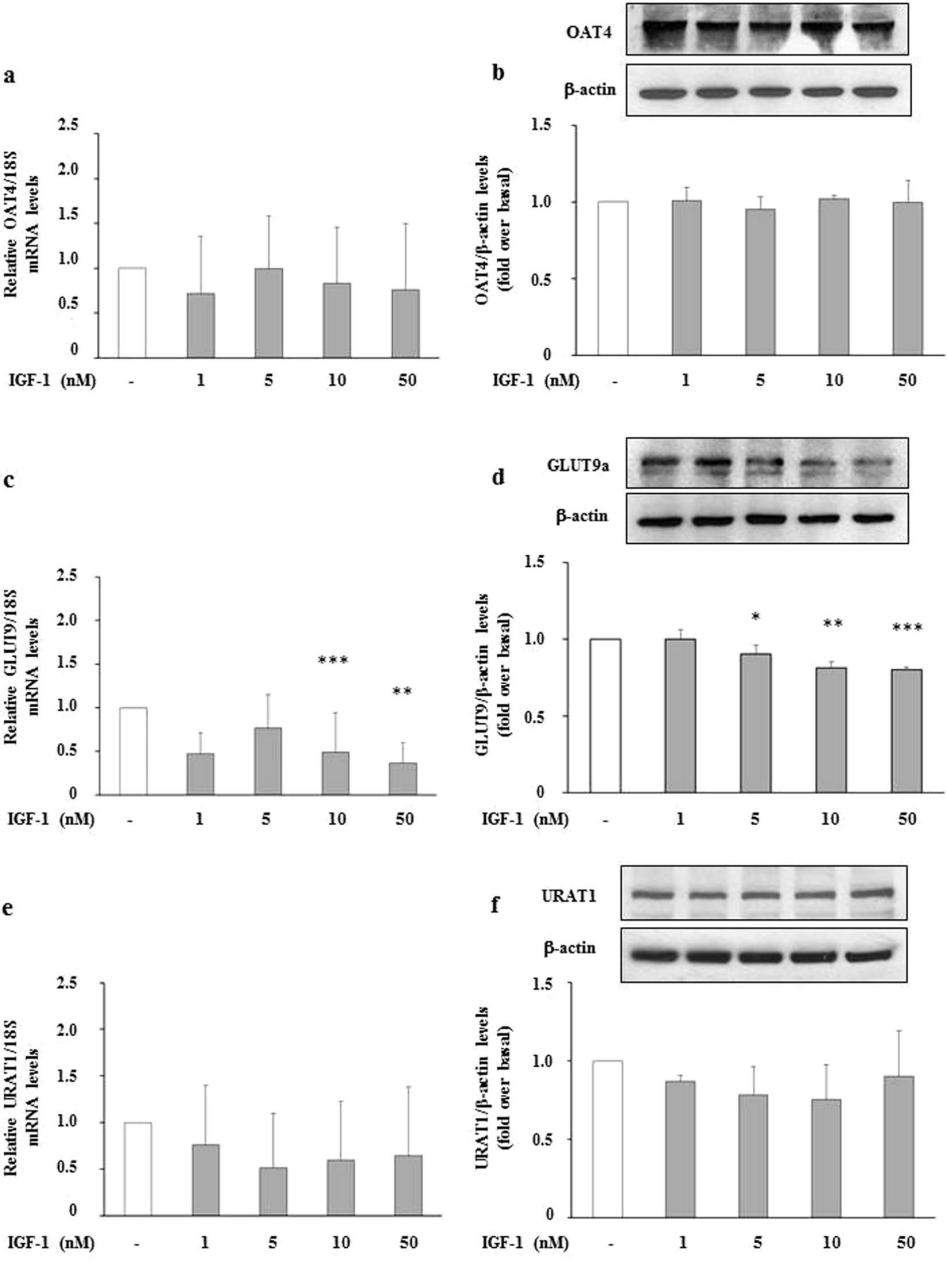
Effect of IGF-1 on urate re-absorptive transporters, mRNA and protein levels. HEK293 cells were incubated in presence of increasing IGF-1 concentrations (5, 10, 50 nM). mRNA levels were measured by Real Time RT-PCR. **(a)** OAT4 mRNA levels; **(b)** OAT4 protein levels; **(c)** GLUT9 mRNA levels; **(d)** GLUT9 protein levels; **(e)** URAT1 mRNA levels; **(f)** URAT1 protein levels. Data are means ± SD of three independent experiments, each done in triplicate. *P < 0.04, **P < 0.02 and ***P < 0.01 vs. basal. To see the original blots from which these images were taken, please refer to the Online Supplementary Information (Supplemental Fig. 2).

To gain insight onto the role of IGF-1 on uric acid transporters, HEK293 cells were incubated with increasing physiologic concentration of IGF-1 (1, 5, 10 and 50 nM) for 24 hours. As shown in Fig. 1, IGF-1 exposure significantly increased mRNA expression of MRP4, NPT1 and BCRP urate transporters (Fig. 1a,c,e, respectively), which mediate uric acid export into urines. The effect of IGF-1 was dose-dependent with maximal effect occurring at 50 nM IGF-1 (*P* < 0.01 for all genes). Likewise, IGF-1 exposure significantly increased protein abundance of MRP4, NPT1 and BCRP uric acid transporters as assessed by Western blot analyses in whole cell lysates (*P* < 0.01 for all transporters) (Fig. 1b,d,f, respectively). Conversely, treatment of HEK293 cells with IGF-1 resulted in a significant reduction in mRNA expression of GLUT9 (Fig. 2c), which is thought to play a major role in the reabsorption of uric acid into the blood and interstitial fluid. Similarly, IGF-1 exposure significantly reduced protein abundance of GLUT9 in whole cell lysates (Fig. 2d). No effect of IGF-1 was observed on OAT4 and URAT1 uric acid transporters expression either at mRNA or protein level (Fig. 2a,b,e,f).

## Discussion

There is evidence from genetic^22,23,32^, and cross-sectional studies^24^ that the IGF-1/IGF-1 receptor signaling pathway may have a role in modulating serum urate levels. Prior *in vitro* and *in vivo* evidences have consistently observed that the genetic variant rs35767, located within the promoter region of *IGF1*, affects the expression levels of IGF-1 and its circulating concentrations, specifically showing that the T allele is the IGF-1 raising polymorphism^26–31^. These findings were the premises which led us to formulate the hypothesis that the rs35767 polymorphism might associate with serum urate concentration. To validate this hypothesis we analyzed over 2500 clinically and metabolically well-characterized Italian adults and we discovered that individuals carrying the TT genotype had significantly reduced serum urate levels when compared with carriers of rs35767 C allele. Furthermore, this association persisted when other factors known to affect urate serum concentration, such as gender, age, BMI, glucose tolerance status, eGFR, and treatment with anti-hypertensive (diuretics, beta-blockers, calcium channel blockers, angiotensin receptor blockers, and ACE inhibitors) were included in the statistical model. Interestingly, subjects carrying the TT genotype displayed higher uricosuria with respect to CT and CC carriers, after adjusting for confounders. This finding coupled with a previous pilot experimental study showing that acute infusion of IGF-1 reduces serum urate levels in healthy individuals^25^ prompted us to hypothesize IGF-1/IGF-1 receptor signaling pathway might contribute to regulate serum urate by modulating expression of uric acid transporters involved in reabsorption and secretion of uric acid in the kidney. In humans, almost all serum urate is initially filtered by the kidney but about 90% is reabsorbed into the blood via a complex interplay of uric acid transporters located along the proximal tubule^35,36^. MRP4, NPT1 and BCRP uric acid transporters are thought to be involved in uric acid excretion, the URAT1 and OAT4 transporters are involved in apical reabsorption of uric acid, while GLUT9, is thought to be the major basolateral uric acid transporter in renal tubular cells contributing to the reabsorption of uric acid into the blood and interstitial fluid. We found, for the first time, that exposure of HEK293 human embryonic kidney cell line to IGF-1 resulted in a dose-dependent increase of MRP4, NPT1 and BCRP uric acid transporters at both mRNA and protein level. By contrast, IGF-1 treatment of HEK293 cells resulted in a significant reduction of GLUT9 expression both at mRNA and protein level. Notably, these results were observed using a range of physiological IGF-1 concentrations (5–50 nM or 38–380 ng/mL), thus suggesting that the present findings may be clinically relevant. Overall, these findings provide a mechanistic explanation of the observed association between serum uric acid levels and the IGF-1-raising rs35767 polymorphism near the promoter region of the *IGF1* gene. However, we cannot rule out the possibility that other mechanisms may account for the observed association between serum uric acid levels and the IGF-1-raising rs35767 polymorphism. For instance, it has been demonstrated that infusion of recombinant IGF-1 increases GFR in normal individuals^25^ and improves renal function in patients with end-stage chronic renal failure^37^. Moreover, IGF-1 circulating levels have been associated with eGFR in both hypertensive individuals^38^ and obese subjects^39^. Thus, the present results may be due to an effect of IGF-1 on renal blood flow and GFR. However, the findings that eGFR did not differ among the genotypes, and the observation that rs35767 genotype was still associated to serum urate concentrations after adjusting for eGFR argue against the possibility.

The current report thrives on the relatively large size of the study cohort, which balances gender, considers a large spectrum of glucose tolerance, and contemplates an ethnically homogeneous Caucasian group. In addition to this, we excluded the presence of chronic conditions potentially affecting both serum uric acid concentration and circulating IGF-1 and the accurate collection of clinical and biochemical information was employed to account for several plausible interferences, together with the centralization of biochemical and analytical assays.

Nonetheless, we need to acknowledge a few limits of our study. Indeed, all biochemical variables, including serum urate concentrations and plasma glucose during OGTT were measured once, and because these measurements are subject to intra-individual variability, we cannot exclude that some imprecision in the proportion of subjects with abnormal glucose tolerance has been introduced. The 24-hour uricosuria measurement solely provides an estimate of the overall output of uric acid; fractional excretion of uric acid would have been a more informative index of its overall tubular handling, but we are not in possession of these data at the moment, and we encourage future research into the renal kinetics of uric acid. Moreover, these results might only be relevant to adult White Europeans, and should not be extended to different ethnicities. Finally, our cohort was enrolled while in the context of hospital care, comprising individuals exposed to different grades of cardiometabolic dysfunctions, which means that the present discoveries should not be generalized to healthy subjects. Collectively, the current findings ought not to be interpreted as final and need replication in other studies and ethnic groups, with independent settings.

In conclusion, these evidences shed new light over the pathophysiological mechanisms connecting the IGF-1 polymorphism rs35767 and serum urate levels. Our genetic investigation strongly demonstrates that a lifelong exposure to lower or higher levels of IGF-1, can modulate uric acid homeostasis at a systemic level. These findings underlie the importance of early intervention directed at increasing IGF-1 levels, expecially in at-risk categories. Such increment can effectively and physiologically be produced through the implementation of a healthy diet plan, exercise and weight loss.

## Electronic supplementary material

Supplementary data

## References

[1] Facchini F, Chen YD, Hollenbeck CB, Reaven GM (1991). Relationship between resistance to insulin-mediated glucose uptake, urinary uric acid clearance, and plasma uric acid concentration. JAMA..

[2] Choi HK, Atkinson K, Karlson EW, Curhan G (2005). Obesity, weight change, hypertension, diuretic use, and risk of gout in men: the health professionals follow-up study. ArchInternMed..

[3] Perticone F (2012). Serum uric acid and 1-h post load glucose in essential hypertension. Diabetes Care..

[4] Choi HK, Ford ES (2007). Prevalence of the metabolic syndrome in individuals with hyperuricemia. Am J Med..

[5] Kodama S (2009). Association between serum uric acid and development of type 2 diabetes. Diabetes Care..

[6] Perticone F (2013). Interaction between uric acid and endothelial dysfunction predicts new onset of diabetes in hypertensive patients. Int J Cardiol..

[7] Zoccali C, Maio R, Mallamaci F, Sesti G, Perticone F (2006). Uric acid and endothelial dysfunction in essential hypertension. J Am Soc Nephrol JASN..

[8] Montalcini T (2007). A.Relation between serum uric acid and carotid intima-media thickness in healthy postmenopausal women. Intern Emerg Med..

[9] Fang J, Alderman MH (2000). Serum uric acid and cardiovascular mortality the NHANES I epidemiologic follow-up study, 1971–1992. National Health and Nutrition Examination Survey. JAMA..

[10] Perticone F (2013). Interaction between uric acid and endothelial dysfunction predicts new onset of diabetes in hypertensive patients. Int J Cardiol..

[11] Sciacqua A (2015). Uric acid is an independent predictor of cardiovascular events in post-menopausal women. Int J Cardiol..

[12] Hediger MA, Johnson RJ, Miyazaki H, Endou H (2005). Molecular physiology of urate transport. Physiology (Bethesda)..

[13] Yang Q (2005). Genome-wide search for genes affecting serum uric acid levels: the Framingham Heart Study. Metabolism..

[14] Nath SD (2007). Genome scan for determinants of serum uric acid variability. J. Am. Soc. Nephrol..

[15] Li, S. *et al*. The GLUT9 gene is associated with serum uric acid levels in Sardinia and Chianti cohorts. *PLoS Genet*. **3**; 10.1371/journal.pgen.0030194 (2007). 10.1371/journal.pgen.0030194 PMC2065883 17997608

[16] Doring A (2008). SLC2A9 influences uric acid concentrations with pronounced sex-specific effects. Nat. Genet..

[17] Vitart V (2008). SLC2A9 is a newly identified urate transporter influencing serum urate concentration, urate excretion and gout. Nat. Genet..

[18] Dehghan A (2008). Association of three genetic loci with uric acid concentration and risk of gout: a genome-wide association study. Lancet..

[19] Kolz, M. *et al*. Meta-analysis of 28,141 individuals identifies common variants within five new loci that influence uric acid concentrations. *PLoS Genet*. **5**, 10.1371/journal.pgen.1000504 (2009). 10.1371/journal.pgen.1000504 PMC2683940 19503597

[20] Yang Q (2010). Multiple genetic loci influence serum urate levels and their relationship with gout and cardiovascular disease risk factors. Circ. Cardiovasc. Genet..

[21] Woodward OM (2009). Identification of a urate transporter, ABCG2, with a common functional polymorphism causing gout. Proc. Natl. Acad. Sci. USA.

[22] Köttgen A (2013). Genome-wide association analyses identify 18 new loci associated with serum urate concentrations. Nat Genet..

[23] Phipps-Green AJ (2016). Twenty-eight loci that influence serum urate levels: analysis of association with gout. Ann Rheum Dis.

[24] Sesti G (2014). Low circulating insulin-like growth factor-1 levels are associated with high serum uric acid in nondiabetic adult subjects. Nutr MetabCardiovascDis..

[25] Guler HP, Schmid C, Zapf J, Froesch ER (1989). Effects of recombinant insulin-like growth factor I on insulin secretion and renal function in normal human subjects. Proc NatlAcad Sci USA.

[26] Canzian F (2006). Polymorphisms of genes coding for insulin-like growth factor 1 and its major binding proteins, circulating levels of IGF-1 and IGFBP-3 and breast cancer risk: results from the EPIC study. Br J Cancer..

[27] Palles C (2008). Identification of genetic variants that influence circulating IGF1 levels: a targeted search strategy. HumMolGenet..

[28] Patel A.V. *et al*. IGF-1, IGFBP-1, and IGFBP-3 polymorphisms predict circulating IGF levels but not breast cancer risk: findings from the Breast and Prostate Cancer Cohort Consortium (BPC3). *PLoSOne* **3**, 10.1371/journal.pone.0002578 (2008). 10.1371/journal.pone.0002578 PMC2440354 18596909

[29] Ollberding NJ (2012). Genetic variants, prediagnostic circulating levels of insulin-like growth factors, insulin, and glucose and the risk of colorectal cancer: the Multiethnic Cohort study. Cancer Epidemiol Biomark Prev..

[30] Mannino G. C. *et al*. A fasting insulin-raising allele at IGF1 locus is associated with circulating levels of IGF-1 and insulin sensitivity. *PloSOne*. **8**, 10.1371/journal.pone.0085483 (2013). 10.1371/journal.pone.0085483 PMC3877361 24392014

[31] Sesti G (2014). A polymorphism at IGF1 locus is associated with carotid intima media thickness and endothelium-dependent vasodilatation. Atherosclerosis..

[32] MacArthur J (2017). The new NHGRI-EBI Catalog of published genome-wide association studies (GWAS Catalog). Nucleic Acids Research..

[33] Marini MA (2017). A polymorphism at IGF1 locus is associated with anemia. Oncotarget..

[34] Levey AS (2003). National Kidney Foundation. National Kidney Foundation practice guidelines for chronic kidney disease: evaluation, classification, and stratification. Ann Intern Med..

[35] Wright AF, Rudan I, Hastie ND, Campbell H (2010). A ‘complexity’ of urate transporters. Kidney Int..

[36] Merriman TR (2015). An update on the genetic architecture of hyperuricemia and gout. Arthritis Res Ther..

[37] Vijayan A, Franklin SC, Behrend T, Hammerman MR, Miller SB (1999). Insulin-like growth factor 1 improves renal function in patients with end-stage chronic renal failure. Am J Physiol..

[38] Perticone F (2009). Insulin-like growth factor-1 and glomerular filtration rate in hypertensive patients. J Hypertens..

[39] Sesti G (2011). IGF-1 levels link estimated glomerular filtration rate to insulin resistance in obesity: A study in obese, but metabolically healthy, subjects and obese, insulin-resistant subjects. Nutr MetabCardiovasc Dis..

