# Supplementary material for: The polymorphism rs35767 at IGF1 locus is associated with serum urate levels
Source: Sci Rep. 2018 Aug 16;8:12255. doi: 10.1038/s41598-018-29665-3 (PMC6095867; doi:10.1038/s41598-018-29665-3)
Supplement: Supplementary file 1 — Supplementary data [file 41598_2018_29665_MOESM1_ESM.pdf]

The polymorphism rs35767 at *IGF1* locus is associated with serum urate levels.

Gaia C. Mannino#[1], Anastasia Fuoco#[1], Maria A. Marini[2], Rosangela Spiga[1], Concetta Di Fatta[1], Elettra Mancuso[1], Francesco Perticone[1], Francesco Andreozzi*[1], Giorgio Sesti[1].

[1]Department of Medical and Surgical Sciences, University "Magna Graecia" of Catanzaro, Italy

[2]Department of Systems Medicine, University of Rome-Tor Vergata, Italy

#These authors have contributed equally to this work

***Corresponding Author** Francesco Andreozzi, Dipartimento Scienze Mediche e Chirurgiche, Università "Magna-Græcia" di Catanzaro, Viale Europa, 88100 Catanzaro (ITALY); Phone:+39-0961-3694327; Fax:+39-0961-3647192; e-mail:andreozzif@unicz.it

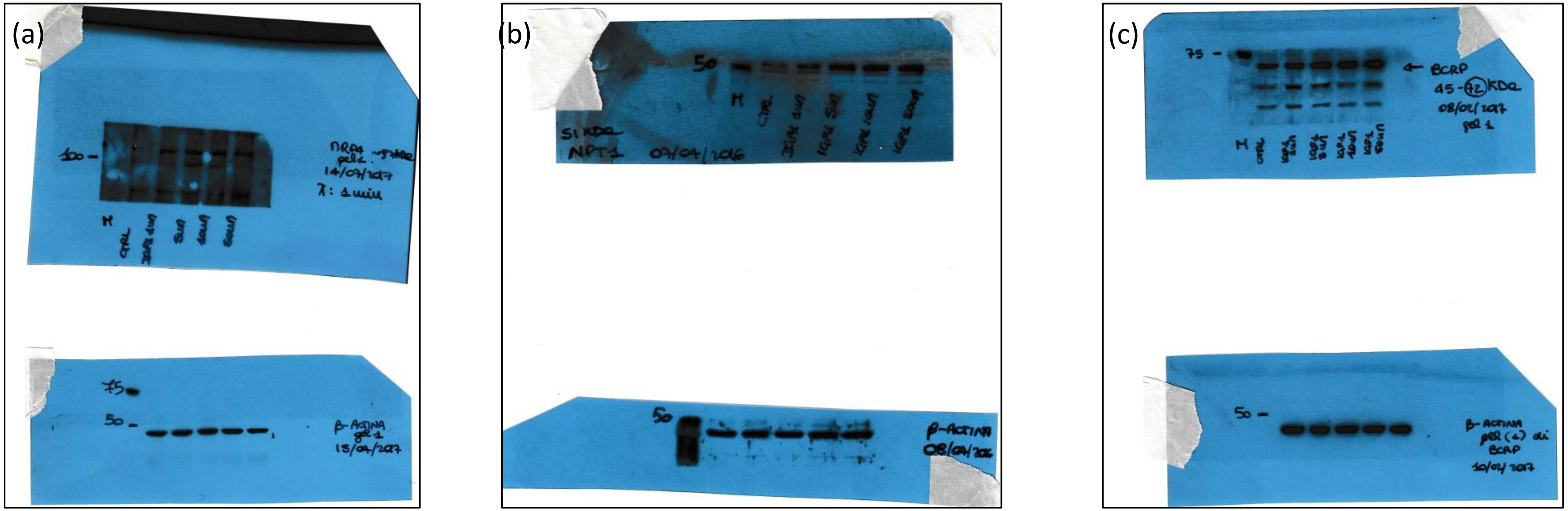

Supplemental figure 1: Effect of IGF-1 on urate excretory transporters. Original Western blot images representing the results of three independent experiments. HEK293 cells were incubated in presence of increasing IGF-1 concentrations (5, 10, 50 nM). **(a)** MRP4 protein levels; **(b)** NTP1 protein levels; **(c)** BCRP protein levels. All data were normalized over beta-actin levels, which are also herein reported.

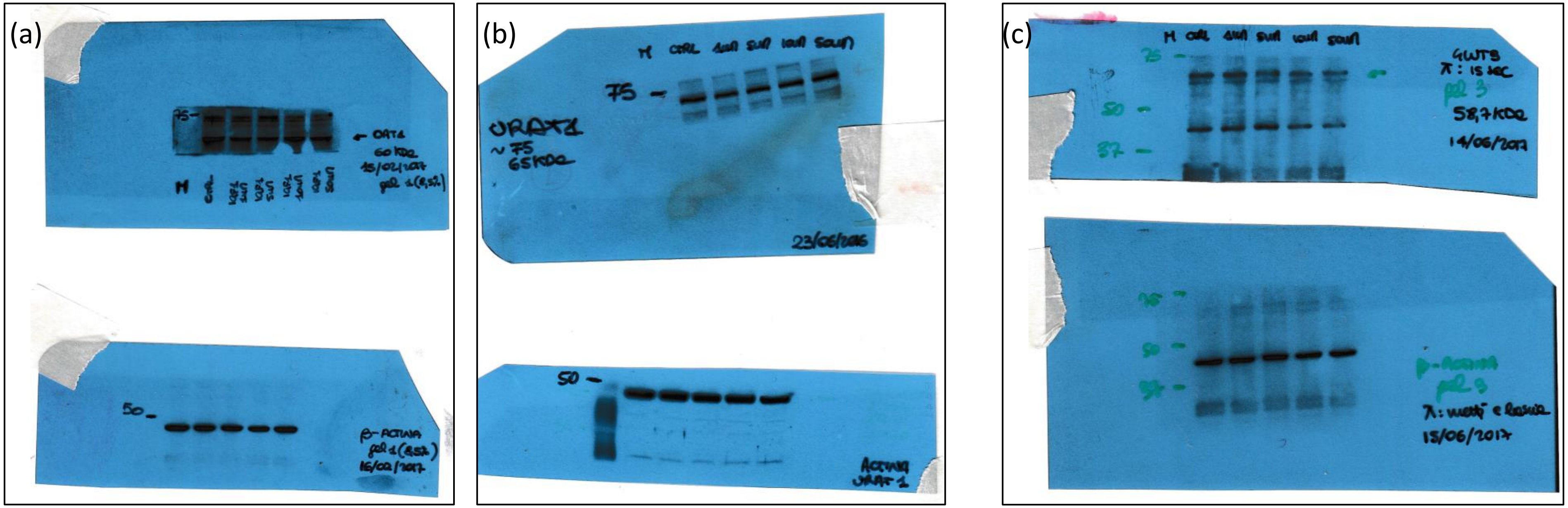

Supplemental figure 2: Effect of IGF-1 on urate re-absorptive transporters. Original Western blot images representing the results of three independent experiments. HEK293 cells were incubated in presence of increasing IGF-1 concentrations (5, 10, 50 nM). **(a)** OAT4 protein levels; **(b)** URAT1 protein levels; **(c)** GLUT9 protein levels. All data were normalized over beta-actin levels, which are also herein reported.
